# Supplementary material for: Modeling glioblastoma heterogeneity as a dynamic network of cell states
Source: Mol Syst Biol. 2021 Sep 16;17(9):e10105. doi: 10.15252/msb.202010105 (PMC8444284; doi:10.15252/msb.202010105)
Supplement: Supplementary file 5 — Source Data for Figure 3 [file MSB-17-e10105-s001.zip › Figure3A_sourcedata/GSEA_3065/hallmarks_state3.GseaPreranked.1623416274744/HALLMARK_P53_PATHWAY.html]

Details for gene set HALLMARK\_P53\_PATHWAY[GSEA]

|  || Dataset | state3 |
| Phenotype | NoPhenotypeAvailable |
| Upregulated in class | na\_neg |
| GeneSet | HALLMARK\_P53\_PATHWAY |
| Enrichment Score (ES) | -0.40534744 |
| Normalized Enrichment Score (NES) | -1.5068351 |
| Nominal p-value | 0.0057971017 |
| FDR q-value | 0.036970407 |
| FWER p-Value | 0.359 |
Table: GSEA Results Summary

  

Fig 1: Enrichment plot: HALLMARK\_P53\_PATHWAY      
 Profile of the Running ES Score & Positions of GeneSet Members on the Rank Ordered List

  

| PROBE | GENE SYMBOL | GENE\_TITLE | RANK IN GENE LIST | RANK METRIC SCORE | RUNNING ES | CORE ENRICHMENT || 1 | APP |  |  | 49 | 0.437 | 0.0228 | No |
| 2 | CD81 |  |  | 109 | 0.337 | 0.0379 | No |
| 3 | SEC61A1 |  |  | 193 | 0.290 | 0.0472 | No |
| 4 | SLC3A2 |  |  | 215 | 0.276 | 0.0628 | No |
| 5 | NOTCH1 |  |  | 272 | 0.253 | 0.0727 | No |
| 6 | CTSD |  |  | 398 | 0.219 | 0.0725 | No |
| 7 | TM7SF3 |  |  | 495 | 0.199 | 0.0744 | No |
| 8 | PLXNB2 |  |  | 742 | 0.165 | 0.0566 | No |
| 9 | PERP |  |  | 798 | 0.158 | 0.0605 | No |
| 10 | TM4SF1 |  |  | 952 | 0.142 | 0.0520 | No |
| 11 | CTSF |  |  | 1103 | 0.129 | 0.0430 | No |
| 12 | CCND2 |  |  | 1286 | 0.116 | 0.0295 | No |
| 13 | F2R |  |  | 1308 | 0.115 | 0.0345 | No |
| 14 | TXNIP |  |  | 1317 | 0.114 | 0.0410 | No |
| 15 | BLCAP |  |  | 1389 | 0.109 | 0.0399 | No |
| 16 | PTPRE |  |  | 1396 | 0.109 | 0.0463 | No |
| 17 | IER5 |  |  | 1491 | 0.103 | 0.0421 | No |
| 18 | SDC1 |  |  | 1568 | 0.100 | 0.0398 | No |
| 19 | RAD9A |  |  | 1614 | 0.097 | 0.0409 | No |
| 20 | POM121 |  |  | 1692 | 0.092 | 0.0380 | No |
| 21 | CD82 |  |  | 1930 | 0.079 | 0.0156 | No |
| 22 | STOM |  |  | 2035 | 0.074 | 0.0084 | No |
| 23 | XPC |  |  | 2135 | 0.071 | 0.0016 | No |
| 24 | CGRRF1 |  |  | 2240 | 0.066 | -0.0062 | No |
| 25 | ABCC5 |  |  | 2250 | 0.066 | -0.0029 | No |
| 26 | HSPA4L |  |  | 2311 | 0.064 | -0.0057 | No |
| 27 | TAP1 |  |  | 2349 | 0.063 | -0.0059 | No |
| 28 | EPHA2 |  |  | 2512 | 0.057 | -0.0210 | No |
| 29 | PTPN14 |  |  | 2681 | 0.051 | -0.0372 | No |
| 30 | SLC7A11 |  |  | 2682 | 0.051 | -0.0339 | No |
| 31 | PIDD1 |  |  | 3011 | 0.041 | -0.0693 | No |
| 32 | SLC35D1 |  |  | 3023 | 0.040 | -0.0679 | No |
| 33 | LDHB |  |  | 3031 | 0.040 | -0.0661 | No |
| 34 | ABAT |  |  | 3059 | 0.040 | -0.0667 | No |
| 35 | RRP8 |  |  | 3129 | 0.038 | -0.0722 | No |
| 36 | ELP1 |  |  | 3248 | 0.035 | -0.0836 | No |
| 37 | SP1 |  |  | 3315 | 0.033 | -0.0892 | No |
| 38 | CCP110 |  |  | 3323 | 0.033 | -0.0878 | No |
| 39 | SERTAD3 |  |  | 3559 | 0.026 | -0.1134 | No |
| 40 | EPHX1 |  |  | 3598 | 0.026 | -0.1161 | No |
| 41 | PLK3 |  |  | 3719 | 0.022 | -0.1286 | No |
| 42 | MDM2 |  |  | 3753 | 0.021 | -0.1310 | No |
| 43 | FUCA1 |  |  | 3766 | 0.021 | -0.1311 | No |
| 44 | RXRA |  |  | 3769 | 0.021 | -0.1299 | No |
| 45 | APAF1 |  |  | 3822 | 0.020 | -0.1347 | No |
| 46 | FOS |  |  | 3899 | 0.018 | -0.1424 | No |
| 47 | CCNK |  |  | 3942 | 0.017 | -0.1462 | No |
| 48 | PCNA |  |  | 3986 | 0.016 | -0.1501 | No |
| 49 | PDGFA |  |  | 3998 | 0.015 | -0.1504 | No |
| 50 | BTG2 |  |  | 4070 | 0.013 | -0.1578 | No |
| 51 | TGFB1 |  |  | 4110 | 0.012 | -0.1615 | No |
| 52 | DNTTIP2 |  |  | 4258 | 0.008 | -0.1781 | No |
| 53 | WRAP73 |  |  | 4514 | 0.001 | -0.2076 | No |
| 54 | KIF13B |  |  | 4592 | -0.001 | -0.2164 | No |
| 55 | TRAFD1 |  |  | 4779 | -0.006 | -0.2377 | No |
| 56 | DRAM1 |  |  | 4865 | -0.008 | -0.2470 | No |
| 57 | PRKAB1 |  |  | 4893 | -0.008 | -0.2496 | No |
| 58 | FOXO3 |  |  | 4960 | -0.010 | -0.2566 | No |
| 59 | PPP1R15A |  |  | 4983 | -0.010 | -0.2585 | No |
| 60 | DDIT4 |  |  | 5025 | -0.012 | -0.2625 | No |
| 61 | STEAP3 |  |  | 5348 | -0.020 | -0.2986 | No |
| 62 | PRMT2 |  |  | 5354 | -0.020 | -0.2979 | No |
| 63 | CYFIP2 |  |  | 5436 | -0.022 | -0.3058 | No |
| 64 | NOL8 |  |  | 5454 | -0.023 | -0.3063 | No |
| 65 | ZNF365 |  |  | 5473 | -0.023 | -0.3069 | No |
| 66 | AEN |  |  | 5589 | -0.027 | -0.3185 | No |
| 67 | IP6K2 |  |  | 5699 | -0.030 | -0.3292 | No |
| 68 | TP53 |  |  | 5700 | -0.030 | -0.3272 | No |
| 69 | BAIAP2 |  |  | 5790 | -0.032 | -0.3354 | No |
| 70 | HDAC3 |  |  | 5820 | -0.033 | -0.3366 | No |
| 71 | RAB40C |  |  | 5849 | -0.034 | -0.3376 | No |
| 72 | BAK1 |  |  | 5895 | -0.036 | -0.3405 | No |
| 73 | RCHY1 |  |  | 5907 | -0.036 | -0.3395 | No |
| 74 | CDKN2AIP |  |  | 5921 | -0.036 | -0.3386 | No |
| 75 | FBXW7 |  |  | 5922 | -0.036 | -0.3362 | No |
| 76 | SESN1 |  |  | 5948 | -0.037 | -0.3367 | No |
| 77 | NDRG1 |  |  | 6040 | -0.041 | -0.3446 | No |
| 78 | RAP2B |  |  | 6137 | -0.044 | -0.3529 | No |
| 79 | ATF3 |  |  | 6159 | -0.045 | -0.3524 | No |
| 80 | VWA5A |  |  | 6165 | -0.045 | -0.3500 | No |
| 81 | PPM1D |  |  | 6214 | -0.047 | -0.3525 | No |
| 82 | ANKRA2 |  |  | 6215 | -0.047 | -0.3495 | No |
| 83 | MAPKAPK3 |  |  | 6223 | -0.047 | -0.3472 | No |
| 84 | PITPNC1 |  |  | 6257 | -0.049 | -0.3479 | No |
| 85 | BMP2 |  |  | 6331 | -0.050 | -0.3530 | No |
| 86 | RALGDS |  |  | 6344 | -0.051 | -0.3511 | No |
| 87 | CSRNP2 |  |  | 6361 | -0.051 | -0.3496 | No |
| 88 | FDXR |  |  | 6438 | -0.054 | -0.3549 | No |
| 89 | HEXIM1 |  |  | 6547 | -0.058 | -0.3636 | No |
| 90 | FAM162A |  |  | 6572 | -0.060 | -0.3625 | No |
| 91 | PLK2 |  |  | 6726 | -0.067 | -0.3759 | No |
| 92 | ITGB4 |  |  | 6730 | -0.067 | -0.3719 | No |
| 93 | DDB2 |  |  | 6772 | -0.070 | -0.3721 | No |
| 94 | MKNK2 |  |  | 6833 | -0.072 | -0.3743 | No |
| 95 | ZFP36L1 |  |  | 6893 | -0.076 | -0.3763 | No |
| 96 | DDIT3 |  |  | 6918 | -0.077 | -0.3740 | No |
| 97 | CCND3 |  |  | 6959 | -0.079 | -0.3735 | No |
| 98 | WWP1 |  |  | 6982 | -0.079 | -0.3709 | No |
| 99 | TPRKB |  |  | 7057 | -0.084 | -0.3740 | No |
| 100 | IRAK1 |  |  | 7071 | -0.085 | -0.3699 | No |
| 101 | TOB1 |  |  | 7088 | -0.086 | -0.3662 | No |
| 102 | RAD51C |  |  | 7264 | -0.098 | -0.3801 | No |
| 103 | PMM1 |  |  | 7437 | -0.111 | -0.3928 | No |
| 104 | RGS16 |  |  | 7541 | -0.122 | -0.3968 | Yes |
| 105 | TSC22D1 |  |  | 7546 | -0.122 | -0.3893 | Yes |
| 106 | CCNG1 |  |  | 7558 | -0.123 | -0.3826 | Yes |
| 107 | JUN |  |  | 7746 | -0.146 | -0.3948 | Yes |
| 108 | TRAF4 |  |  | 7838 | -0.156 | -0.3952 | Yes |
| 109 | PHLDA3 |  |  | 7839 | -0.157 | -0.3850 | Yes |
| 110 | HMOX1 |  |  | 7843 | -0.157 | -0.3750 | Yes |
| 111 | TAX1BP3 |  |  | 7895 | -0.165 | -0.3702 | Yes |
| 112 | EI24 |  |  | 7965 | -0.179 | -0.3665 | Yes |
| 113 | MXD4 |  |  | 7990 | -0.183 | -0.3574 | Yes |
| 114 | ZMAT3 |  |  | 8086 | -0.201 | -0.3553 | Yes |
| 115 | NINJ1 |  |  | 8160 | -0.218 | -0.3495 | Yes |
| 116 | BTG1 |  |  | 8162 | -0.218 | -0.3354 | Yes |
| 117 | UPP1 |  |  | 8293 | -0.256 | -0.3338 | Yes |
| 118 | GADD45A |  |  | 8306 | -0.260 | -0.3182 | Yes |
| 119 | TRIAP1 |  |  | 8318 | -0.262 | -0.3024 | Yes |
| 120 | DCXR |  |  | 8321 | -0.264 | -0.2854 | Yes |
| 121 | HRAS |  |  | 8396 | -0.293 | -0.2749 | Yes |
| 122 | RACK1 |  |  | 8406 | -0.297 | -0.2566 | Yes |
| 123 | BAX |  |  | 8408 | -0.299 | -0.2372 | Yes |
| 124 | RPL18 |  |  | 8424 | -0.304 | -0.2192 | Yes |
| 125 | RPS27L |  |  | 8528 | -0.351 | -0.2082 | Yes |
| 126 | ISCU |  |  | 8531 | -0.352 | -0.1854 | Yes |
| 127 | SAT1 |  |  | 8533 | -0.354 | -0.1625 | Yes |
| 128 | CDKN1A |  |  | 8657 | -0.461 | -0.1467 | Yes |
| 129 | RPL36 |  |  | 8694 | -0.512 | -0.1175 | Yes |
| 130 | HINT1 |  |  | 8712 | -0.547 | -0.0838 | Yes |
| 131 | S100A10 |  |  | 8717 | -0.558 | -0.0478 | Yes |
| 132 | RPS12 |  |  | 8747 | -0.792 | 0.0005 | Yes |
Table: GSEA details [plain text format]

  

Fig 2: HALLMARK\_P53\_PATHWAY: Random ES distribution      
 Gene set null distribution of ES for **HALLMARK\_P53\_PATHWAY**
